# Determination of Minimum Miscibility Pressure of CO_2_–Oil System: A Molecular Dynamics Study

**DOI:** 10.3390/molecules26164983

**Published:** 2021-08-17

**Authors:** Ding Li, Shuixiang Xie, Xiangliang Li, Yinghua Zhang, Heng Zhang, Shiling Yuan

**Affiliations:** 1 State Key Laboratory of Petroleum Pollution Control, CNPC Research Institute of Safety & Environment Technology, Beijing 100000, China; blyceoh@163.com (D.L.); tu_95ms@sina.com (S.X.); 2 Key Lab of Colloid and Interface Chemistry, Shandong University, Jinan 250100, China; shilingyuan@sdu.edu.cn; 3 Shengli Oil Field Exploration and Development Research Institute, Dongying 257000, China; lxliang1964@163.com (X.L.); zhangyh3000@foxmail.com (Y.Z.)

**Keywords:** minimum miscible pressure, CO_2_ enhanced oil recovery, molecular dynamics

## Abstract

CO_2_ enhanced oil recovery (CO_2_-EOR) has become significantly crucial to the petroleum industry, in particular, CO_2_ miscible flooding can greatly improve the efficiency of EOR. Minimum miscibility pressure (MMP) is a vital factor affecting CO_2_ flooding, which determines the yield and economic benefit of oil recovery. Therefore, it is important to predict this property for a successful field development plan. In this study, a novel model based on molecular dynamics to determine MMP was developed. The model characterized a miscible state by calculating the ratio of CO_2_ and crude oil atoms that pass through the initial interface. The whole process was not affected by other external objective factors. We compared our model with several famous empirical correlations, and obtained satisfactory results—the relative errors were 8.53% and 13.71% for the two equations derived from our model. Furthermore, we found the MMPs predicted by different reference materials (i.e., CO_2_/crude oil) were approximately linear (R^2^ = 0.955). We also confirmed the linear relationship between MMP and reservoir temperature (T_R_). The correlation coefficient was about 0.15 MPa/K in the present study.

## 1. Introduction

Global warming has caused great changes such as continued sea level rise, which is irreversible over hundreds to thousands of years. CO_2_ is the culprit of this phenomenon. CCUS (CO_2_ capture, utilization, and storage) is a new technology developed from CCS (CO_2_ capture and storage) that can bring economic benefits while reducing CO_2_ emissions and alleviating global warming [1]. CO_2_ enhanced oil recovery (CO_2_-EOR) is one of the effective ways of CCUS. The captured CO_2_ is squeezed into the oil reservoirs that have been exploited, and the interaction between CO_2_ and crude oil is used to improve the properties of the crude oil, thereby displacing more crude oil from the crust [2]. Research has shown that CO_2_-EOR can improve crude oil recovery significantly and extend the life of oil reservoirs [3,4]. Hence, CO_2_-EOR has been fundamentally well researched in laboratories and applied in industries as an efficient approach since the 1970s [5].

There are two different miscible and immiscible states in CO_2_-EOR. Under the former condition, CO_2_ and crude oil can completely integrate into one phase, resulting in a much higher recovery rate than the latter. For the former, there is a minimum pressure above which CO_2_ and crude oil can be miscible. This minimum pressure value, also called the minimum miscible pressure (MMP), is a vital parameter in the process of CO_2_-EOR. Nevertheless, considering the massive influencing factors, the accurate determination of MMP remains a major challenge [6].

To date, there are various ways to predict MMP such as experimental measurement and computational methods. The former has been widely used due to their high precision. Within them, slim-tube experiments [7,8,9], as a necessary test in the industry, is considered to be the standard experimental procedure. Rising-bubble apparatus (RBA) [10,11] and vanishing interfacial tension (VIT) [12,13,14,15] are also frequently utilized to determine MMP because of their simplicity and flexibility. Although these experimental measurements have accurate techniques, they still suffer from some disadvantages including time-consumption and operation cost. Furthermore, it is difficult for any experimental method to simulate the real conditions of the crude oil reservoirs completely so that their results are greatly influenced by the instruments.

The application of computational techniques is an available alternative approach to experiments. In 1960, the first empirical MMP correlation was proposed by Benham et al. [16]. The reported equation was correlated using three pseudo-components presenting a multi-components system, and some satisfactory results were obtained based on this model. Thereafter, an increasingly number of correlations were developed for MMP prediction [17,18,19,20]. Researchers found that the more useful parameters an equation used, the better performance the model had [21]. These parameters generally included reservoir temperature (T_R_), composition of drive gas (CO_2_, H_2_S, N_2_, and C_1_–C_5_), molecular weight of C_5+_ fraction in crude oil (MW_c5+_), and the ratio of volatile (C_1_ and N_2_) to intermediate (C_2_–C_4_, H_2_S, and CO_2_) in crude oil (Vol./Int.).

In addition to the conventional empirical formula models, the parameters above are often used in some intelligent algorithms based on machine learning. For instance, artificial neural networks (ANNs) can learn from large amounts of input data, and reflect their relationships more effective than conventional techniques [22]. Determination of network structure and its parameters are two crucial steps in achieving high performance from ANN. One part of the data is used to train and look for a suitable structure and optimal parameters, while the other tests the prediction accuracy of the model. Based on the principle, back propagation (BP) [23] and radial basis function (RBF) [24] are proposed. Beyond that, a series of optimization methods such as genetic algorithm (GA) [25], particle swarm optimization (PSO) [26], support vector machine (SVM) [27], and hybrid-ANFIS [28] have also been developed for MMP determination. In a previous study [29], we compared four estimation methods and found that the machine learning intelligent algorithm had a higher precision to the MMP than pure linear model. In addition, some reports that combined multiple approaches showed better results [30,31,32,33,34].

However, all of the above methods cannot give a direct explanation of the MMP from a microscopic view. They are all based on the existing oilfield data, which means that the established model will inevitably be affected by specific situation. To put it another way, these methods can be considered as pure mathematical statistics methods that have low levels of universality for different CO_2_-EOR.

**Figure 1:**
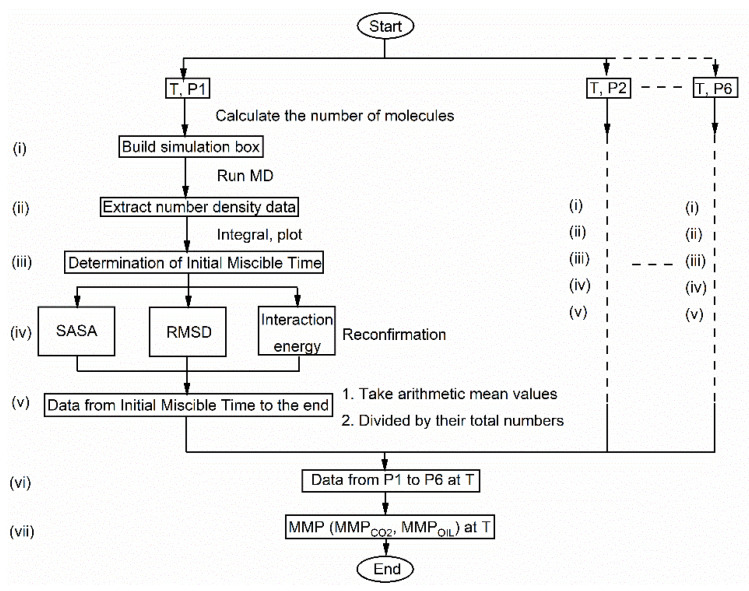
Flowchart of proposed MMP prediction model. (i) Construction of the simulation system, (ii) Extracting number density data after MD simulation, (iii) Determination of initial miscible time, (iv) Reconfirmation of initial miscible time, (v) Processing data from initial miscible time to the end, (vi) Processing data from P1 to P6 at T, (vii) Acquisition of MMP.

Against this backdrop, the current study proposes a novel MMP prediction model at the molecular level, and the research process was not affected by other external objective factors. Therefore, the model represents a new strategy. First, we built a simulation box that contained CO_2_ and crude oil with an obvious phase interface. To mimic the contact between CO_2_ and crude oil, these molecules were gradually mixed until they were miscible with time evolution by using molecular dynamics. After calculating the ratio of CO_2_/crude oil atoms that passed through the initial interface, we found the connection between the ratio value with the miscible state. When the ratio changes from decreasing to stable, it indicates that the system has entered a miscible state, while the pressure corresponding to the inflection point is MMP. Figure 1 is a flow chart that shows all the main steps of modeling. The main objective of this study was to reveal the principle of the MMP formation at the molecular level and provide more feasible ideas for the prediction of the MMP.

## 2. Simulation Method

### 2.1. Simulation and Force Field

The molecular dynamics simulation was performed by the GROMACS 4.6.7 package [35,36], and AMBER 03 all-atom force field [37]. Parameters set for all components of crude oil and CO_2_ were generated from Automated Topology Builder and Repository databases [38,39].

The convergence criterion of energy minimization was 1000 kJ/(mol·nm). In the simulation, a velocity rescaling thermostat with a 0.1 ps time constant was selected as the temperature coupling method [40]. Berendsen pressure coupling with 1.0 ps time constant was selected as the pressure coupling method. The isothermal compression factor was set to 4.5 × 10^−5^ bar^−1^ [41]. The time step was 2 fs, and periodic boundary conditions were applied in the XY directions [42]. Walls were set at the top and bottom of the Z-direction in the simulated box to ensure that all atoms passed through the initial interface to achieve the miscibility. Bond lengths were constrained by the LINCS algorithm [43]. During the simulation, van der Waals interactions with the Lennard–Jones potential was cut off at 1.4 nm. Coulomb interaction used the particle-mesh Ewald summation method [44,45]. The Verlet list was updated every 10 steps. The Maxwell–Boltzmann distribution was employed to set the initial atomic velocities of the systems [46]. The trajectories were integrated by the leapfrog Verlet algorithm [47].

### 2.2. Simulation System

In a real situation, the chemical components of crude oil are highly complex. Under the current experimental conditions, it is time-consuming to precisely analyze the exact constitution of its components. In order to get as close as possible to the real situation, the oil model was designed based on Miranda’s works [48,49], which were used to explore the interface properties between crude oil and different fluids. Their model contained alkanes (72 hexane, 66 heptane, 78 octane, and 90 nonane molecules), cyclanes (48 cyclohexane and 78 cycloheptane molecules), and aromatics (30 benzene and 78 toluene molecules), and has been proven reliable by Song et al. [50].

At 333 K and 10 MPa, all alkanes, cyclanes, and aromatics were added into a cubic box (x = 9 nm, y = 9 nm, z = 9 nm) randomly. Then, energy minimization was performed to eliminate opposed-conformation. In order to mimic the state of crude oil in the reservoir, we performed a 30 ns NPT ensemble simulation to obtain its equilibrium state. After equilibration, the size of simulation box changed to 5.2 nm × 5.2 nm × 5.2 nm.

**Figure 2:**
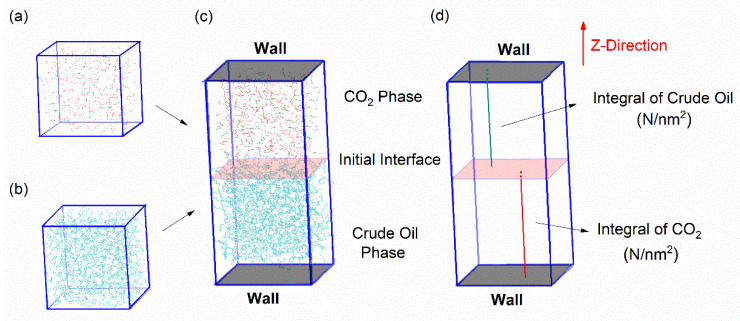
Construction of the simulation system. (**a**) CO_2_ phase. (**b**) Crude oil phase. (**c**) Initial simulation box. (**d**) Integral of CO_2_/crude oil molecules passing through the initial phase interface.

Furthermore, we built a box of the same size, stochastically adding 561 CO_2_ molecules to mimic the supercritical CO_2_ fluid (333 K, 10 MPa). Energy minimization and 30 ns NVT ensemble simulation enabled the CO_2_ to reach its equilibrium state. To simulate the contact between CO_2_ and crude oil, the two boxes were integrated into one rectangular simulation box, and the height of new box in the Z-direction was slightly increased to 11.2 nm to avoid intermolecular overlap, as shown in Figure 2. After that, at least 10 ns NPT ensemble MD simulation was performed.

## 3. Results and Discussion

In the last NPT ensemble simulation, the size of the box gradually stabilized over time. When CO_2_ was miscible with crude oil, the NPT ensemble achieved equilibrium and the box size remained unchanged. However, the change in the size of the box cannot intuitively reflect the miscibility process. Therefore, we introduced the number density of CO_2_/crude oil.

The product of number density and volume of a box is the total number of atoms. When the system achieves equilibrium, the ratio of CO_2_/crude oil atoms in the upper or lower half of the box should be 50%. In our simulation system, the Z-direction height will not change due to the presence of walls. Therefore, the integral change in the number density in the Z-direction (i.e., the integral bars in Figure 2d) can reflect the change in the size of the box and further reflect the mixing progress. When the system achieves equilibrium, the integral of the number density in Z-direction will also be constant.

### 3.1. Definition of Initial Miscible Time

First, the initial miscible time was defined. It refers to the moment when CO_2_ and the crude oil phases just reach the miscible state during their mixing progress, and they can keep the miscible state afterward. The purpose is to ensure that data after this time are miscibility data. We used the CO_2_ phase as an example to illustrate the calculations. Its number density data were extracted along the Z-direction of the box from 0.5 ns to 10.0 ns every 0.5 ns after the NPT ensemble was run. Hence, there were 20 sets of data in total. We can obtain the number of CO_2_ atoms in the lower half of the box by integrating the density of CO_2_ along the Z-direction below the initial interface at each cut-off time. Since CO_2_ was not distributed in the box below the initial interface at the beginning, the integral values we obtained corresponded to the number of CO_2_ atoms passing through the initial interface at the cut-off time. More vividly, it is an integral bar in the three-dimensional space of the box along the Z-direction, as shown in Figure 2d.

**Figure 3:**
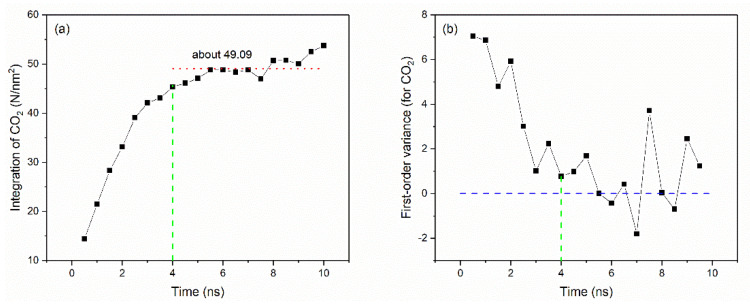
The number of CO_2_ atoms passing through the initial interface (**a**) and its first–order variance (**b**) with time evolution (333 K, 10 MPa).

Furthermore, a curve of the number of CO_2_ atoms passing through the initial interface over time can be plotted. Figure 3 shows the change in the number of CO_2_ atoms in the lower half of the box at 333 K and 10 MPa: it gradually increased from zero to a stable value (about 49.09), and then tended to be stable. It is worth noting that each molecule always kept in continuous random motion, thus it is normal to have positive and negative fluctuations after miscibility. For the selection of the initial miscibility time, the establishment standard is to find the time when the curve becomes stable and the change is very gentle after the miscibility reference line (i.e., the 49.09 line in Figure 3a). This is also the time when the miscibility has just been achieved. The first-order variance of data with time evolution (Figure 3b) reflects the trend of data changes more intuitively. It should be noted that it is not the “initial miscibility” that is already zero, but the time corresponding to the point relatively close to zero. Based on the situation in Figure 2 and Figure 3, it can be guaranteed that the time at 4 ns: (i) the vertical axis value is already very close to the reference line, and (ii) the curve’s upward trend has slowed down.

Based on similar treatments, crude oil atoms passing through the initial interface with time evolution (Figure S1) and its first-order variance (Figure S1b) can also be drawn. Figure 3 and Figure S1 show that the changes in CO_2_ and crude oil were quite similar. Combined with the analyses above, we can preliminarily conclude that 4 ns is the initial miscible time, and is also the time when the CO_2_–oil system achieved miscibility.

### 3.2. Reconfirmation of Initial Miscible Time

#### 3.2.1. Solvent Accessible SURFACE Area (SASA) Analysis

**Figure 4:**
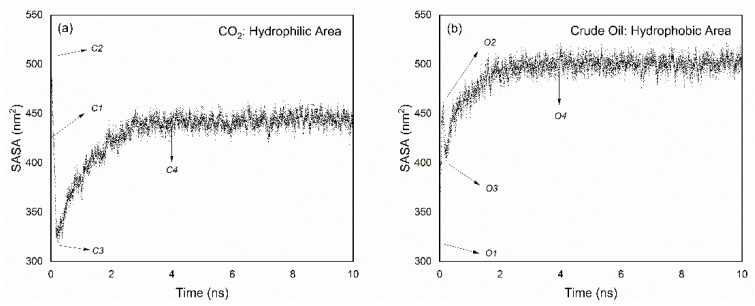
SASA analysis for CO_2_ (**a**) and crude oil (**b**).

To confirm the initial miscible time discussed in previous parts, solvent accessible surface area (SASA) was calculated. SASA represents the hydrophobic, hydrophilic, and total solvent accessible surface area for each component of the simulated system. Figure 4 shows the change in the hydrophilic area of CO_2_ from 0 ns to 10 ns and went through roughly three processes: (i) At the beginning, SASA increased rapidly and reached the highest point (from C1 to C2); (ii) SASA dropped to the lowest point in a short period of time (from C2 to C3); and (iii) SASA gradually rose to basic stability and attained a state of dynamic balance (from C3 to C4, and the time was after about 4 ns).

C1 and C2 are adjustments to the initial configuration in the molecular dynamics NPT ensemble, which was not our focus. This can be contributed to the molecular dynamic method being able to readjust the molecular conformation in the model under the NPT ensemble, and we focused more on the change in conformation after being readjusted. With the blending of CO_2_ and crude oil phases, both gradually achieved the best coexistence state (after C4).

Similarly, the changing trend of hydrophobic surface area of crude oil can be obtained by the same method. As shown in Figure 4b, a similar SASA change was observed in which the area increased and then decreased rapidly with time evolution (from O1 to O3 in Figure 4b). From Figure 4, it is reasonable to select 4 ns as the initial miscible time, and the data after 4 ns can be used to discuss the miscibility.

#### 3.2.2. Root Mean Square Deviation (RMSD) Analysis

**Figure 5:**
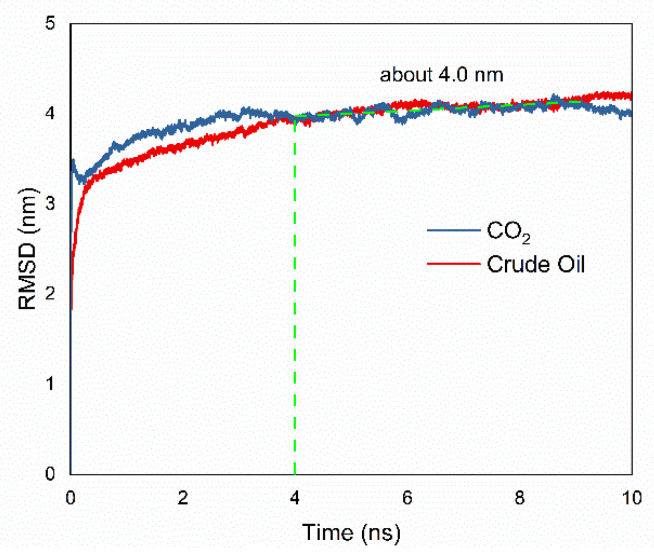
RMSD analysis for CO_2_ and crude oil.

Root mean square deviation (RMSD) compares each molecular structure in the simulation from the trajectory to the initial reference structure, reflects the change in its conformation, and is calculated by Equation (1).

(1) $$RMSD=\sqrt{\frac{1}{N}\sum_{i=1}^{N}{\left(\left|r_{i}\left(t\right)-r_{i}\left(0\right)\right|\right)}^{2}}$$

where *N* is the total number of atoms (CO_2_/crude oil); and *r_i_*(0) and *r_i_*(t) are the initial position and the position of atom *i* at time *t.* Figure 5 displays the RMSD of CO_2_/crude oil during NPT ensemble as a function of time. It is interesting to note that CO_2_ has a higher RMSD value than crude oil at the beginning, which indicates that CO_2_ has better mobility. From 4 ns to 10 ns, the RMSD of CO_2_/crude oil in the box fluctuated with time evolution. Both were gathered around 4 nm of RMSD, which signifies that the system achieved equilibrium after 4 ns.

#### 3.2.3. Interaction Energy Analysis

**Figure 6:**
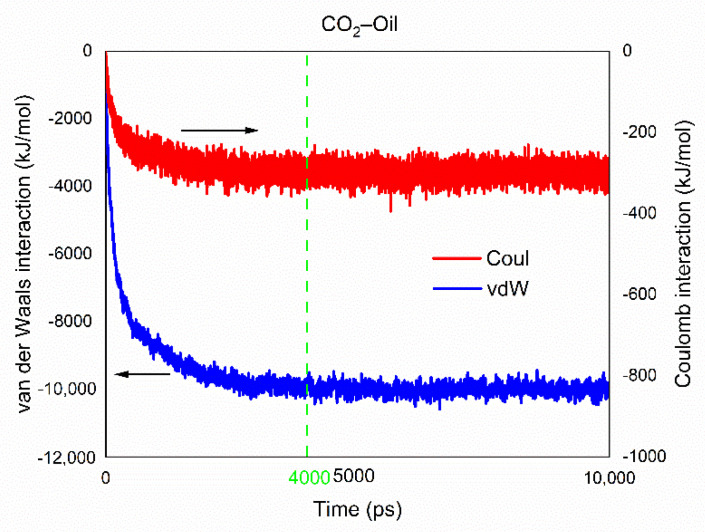
Interaction energy analysis.

The energy changes can reveal the changes in conformation in the simulated system and represent the miscibility process between CO_2_ and crude oil phases. Interaction energy is a type of non-bonding interaction including long-range Coulomb interaction and short-range van der Waals interaction. As shown in Figure 6, the system was dominated by van der Waals interaction, while Coulomb interaction accounts for only about one-tenth of the former. This is because both CO_2_ and crude oil are non-polar molecules and do not have forces such as strong hydrogen bonding interaction. The intermolecular forces are mainly dispersive forces. The dispersion forces increased with time evolution, and the van der Waals potential energy and the total intermolecular potential energy increased accordingly.

When the system achieved equilibrium, the total interaction energy between CO_2_ and crude oil also reached its maximum and remained dynamically stable. Figure 6 clearly indicates that van der Waals interaction and Coulomb interaction both remained stable after 4 ns.

### 3.3. Acquisition of MMP

Once the initial miscible time system at 333 K and 10 MPa has been successfully determined, the number of CO_2_ atoms in the lower half of the box and crude oil atoms in the upper half of the box after 4 ns were taken as the arithmetic mean respectively. It needs to point out that the number of CO_2_ molecules under different pressures are different for 333 K system (Table S1). In order to reflect the general laws, the ratio of mean value to their respective total number of CO_2_ and crude oil atoms in the box was calculated. Similarly, the ratio of CO_2_/crude oil passing through the initial interface to their respective totals at 333 K for 15 MPa, 20 MPa, 25 MPa, 30 MPa, and 35 MPa were also calculated, as shown in Table 1.

From 10 to 35 MPa, the data of ratio decreased first and then became stable. We believe that this is because the system reached its peak pressure at 333 K. When the system exceeded this pressure, the additional simulation will not affect the value of each ratio. Therefore, the pressure is the theoretical MMP at 333 K.

**Figure 7:**
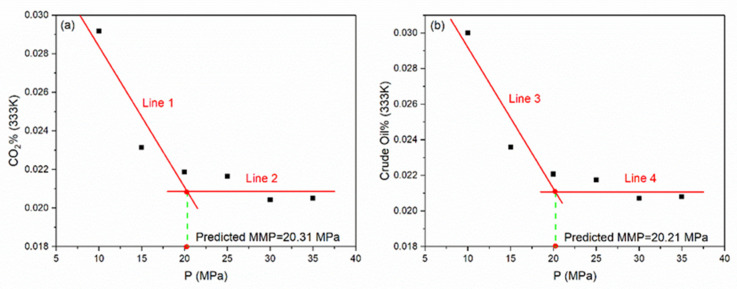
Acquisition of MMP (333 K) for CO_2_ (**a**) and crude oil (**b**).

For the sake of confirming the MMP, we handled the data according to its regularity. The first three decreasing points were fitted linearly, representing the systems before MMP, and an equation in the form of y = kx + b was obtained. The last three nearly equal points were regarded as stable points, representing the systems after MMP, thus, another equation of y = x can be acquired by taking their arithmetic mean. We can subsequently obtain an intersection point as a consequence of simultaneous equations, and the abscissa corresponding to this point is the exact MMP at 333 K. As shown in Figure 7a,b, it was 20.31 MPa for CO_2_ and 20.21 MPa for crude oil.

### 3.4. MMP in Different Temperature Systems

We continued to simulate and analyze the data under the condition of 343 K, 353 K, 363 K, and 373 K at 10 MPa, 15 MPa, 20 MPa, 25 MPa, 30 MPa, and 35 MPa, respectively. It is worth pointing out that the density of CO_2_ varies greatly at different temperatures and pressures, therefore we computed the number of CO_2_ molecules under different conditions. The amount of CO_2_ molecules added to each simulation system are listed in Table S1. Afterward, a summary of initial miscible time in different systems can be obtained according to the methods in Section 3.1 and Section 3.2, as listed in Table 2. Table S2 summarized all the integral values in this study.

The ratio of CO_2_ and crude oil atom numbers that passed through the initial interface to their respective totals when they achieved miscibility can be obtained. Consequently, MMP of 343 K, 353 K, 363 K, and 373 K were obtained according to the method described in Section 3.4 by plotting and curve fitting (Figure S2). Table 3 summarizes the results.

### 3.5. Model Assessment

**Figure 8:**
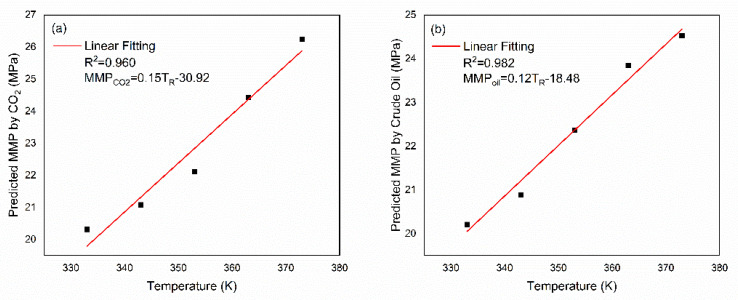
Relationships between T_R_ and MMP for CO_2_ (**a**) and crude oil (**b**).

We fitted the MMP obtained from CO_2_/crude oil to T_R_, respectively, and obtained two prediction equations (Figure 8). It can be compared with the experimental results to check the predictive performance of the model. Recently, Yu et al. used a combination method of slim-tube experiments and interfacial tension (IFT) to perform MMP measurements on tight oil from the Long Dong region of the Ordos Basin. This method has higher credibility than slim-tube experiments [51]. Afterward, we compared our model with several famous empirical correlations to illustrate its accuracy by employing the experimental method proposed by Yu et al. as the benchmark. Table 4 reports the relative error. Details of these empirical correlations are summarized in Table S3.

The relative error obtained from crude oil was similar to Lee [52], and the CO_2_ relative error was similar to Alston et al. [53]. The equation proposed by Shokir [54] was based on an alternating conditional expectation algorithm, and had a relative error of 11.89 %. The model of Emera and Sarma [25] can be employed to calculate the MMP of impure CO_2_ injection, but has poor accuracy. Beyond that, the performances of Cronquist [55], Glaso [56], and Yellig and Metcalfe [57] were also unsatisfactory. The overall results can prove that even if only the influencing factor of T_R_ is considered, the model proposed in this study had satisfactory prediction accuracy.

### 3.6. Comparison of MMP Predicted by CO_2_ and Crude Oil

**Figure 9:**
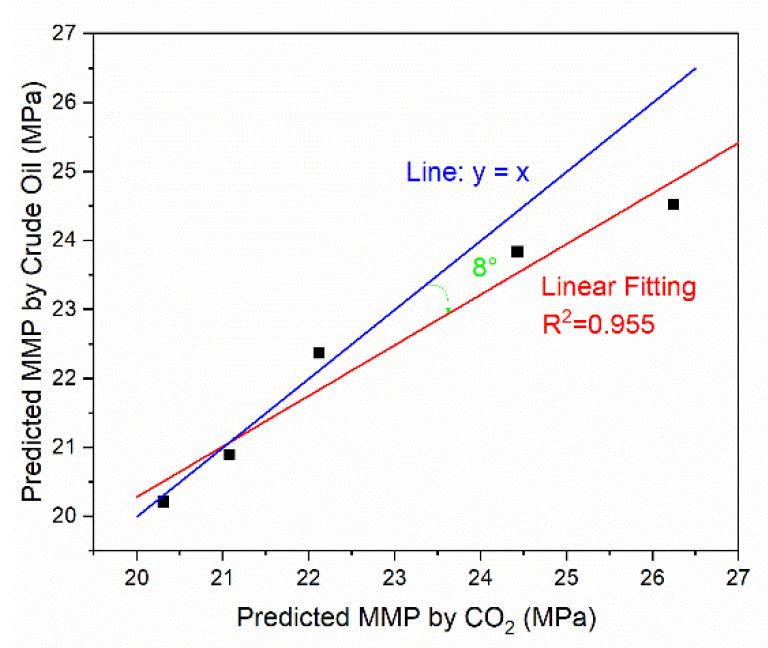
Comparison of MMP predicted by CO_2_ and crude oil.

The relationships between MMP predicted by CO_2_ and crude oil can be compared. It is more intuitive to reflect the data in Table 3 to Figure 9. In Figure 9, the blue line represents the curve whose analytical formula is y = x, and the red line is the fitting curve for the data. It can be found that the MMPs predicted by CO_2_ and crude oil were approximately linear (R^2^ = 0.955). Furthermore, in the same simulation system, the MMP values obtained from different reference materials (CO_2_/crude oil) were not identical as there was a slight difference between them (i.e., an included angle of about 8°).

### 3.7. Relationships between T_R_ and MMP

However, the real situation of each oil reservoir varies, and the composition of injected gases is also different in EOR, so it is meaningless and almost impossible to obtain the accurate relationship between each influence factor and MMP. For a certain influencing factor, we can explore the qualitative relationship between the factor and MMP. Oil reservoir temperature (T_R_) is usually regarded as one of the most important factors affecting MMP [58]. Exploring the influence of T_R_ on MMP is the core of many studies (such as the fitting of empirical formula). Recently, Zheng et al. [59] proposed a novel oil droplet volume measurement method (ODVM) to measure the multiple contact minimum miscibility pressure (MCMMP) and first contact miscibility pressure (FCMP) in the CO_2_/n-hexadecane (C_16_H_34_) and CO_2_/liquid paraffin systems. Their experimental data showed that the measured MMP values of two CO_2_–oil systems increased linearly with T_R_. Furthermore, Mostafa et al. found that the MMP is a linear function of temperature with a slope of 0.15 MPa/K [60].

The modeling method of this study shows that the relationship between T_R_ and MMP can be identified in the principle of miscibility because it is not affected by other external objective factors. As shown in Figure 8, for both CO_2_ and crude oil, the change in T_R_ and MMP basically conformed to a linear relationship, thus a fairly good fitting result can be obtained by using the first-order linear equation. This is because the increase in T_R_ can effectively reduce the solubility of CO_2_ in crude oil, which is not conducive to the mixing progress of CO_2_ and crude oil, ultimately leading to the increase in MMP. During the temperature range (333–373 K), it is a linear change with a slope of 0.15 MPa/K and 0.12 MPa/K and consistent with the experimental results.

## 4. Conclusions

In this paper, a novel molecular dynamics-based model to determine minimum miscible pressure of CO_2_–oil system was developed. The model characterized the miscible state by calculating the ratio of both CO_2_ and crude oil atoms that passed through the initial interface to their respective totals. These ratio values dropped rapidly and fluctuated after a certain value with the increase in pressure at a fixed T_R_. The value is the MMP of T_R_. In comparison with conventional prediction approaches, the present work proposed a straightforward model to simulate the complex miscibility of CO_2_ and crude oil, and the miscible principle was clarified at the molecular scale.

Based on the above studies, the newly proposed model is believed to be reliable for the prediction of MMP. However, there still remain some distinctions when compared to the real situation, which may have a certain impact on the prediction [61]. We have begun to adjust the model to enhance its application. For example, we plan to introduce silica slab and asphaltenes to mimic the real situation of crust and heavy oil, respectively. To sum up, the following conclusions can be drawn:

(1) The molecular scale mixing progress of CO_2_ and crude oil was investigated in principle for the first time, and the research process was not affected by other external objective factors. Results showed that the ratio of CO_2_/crude oil atoms that passed through the initial interface to their respective totals was always the same when the system was miscible. The proposed model had good prediction capabilities.
(2) In the process of the simulation, the SASA, RMSD, and interaction energy of CO_2_/crude oil changed obviously, thus they can be used as criteria of miscibility between both phases.
(3) The MMP predicted by CO_2_ of the CO_2_–oil system were 20.31 MPa, 21.08 MPa, 22.12 MPa, 24.43 MPa, and 26.25 MPa at temperatures of 333 K, 343 K, 353 K, 363 K, and 373 K, respectively, and MMPs predicted by crude oil were 20.21 MPa, 20.89 MPa, 22.36 MPa, 23.84 MPa, and 24.52 MPa at the same temperatures. The two sets of data had a linear relationship.
(4) MMP and reservoir temperature (T_R_) had a linear relationship in the present work, and the slope was about 0.15 MPa/K, which are in agreement with theoretical analyses and literature results.

## Figures and Tables

**Table 1:** The ratio of CO_2_ and crude oil atoms passing through the initial interface to their respective totals.

	CO_2_	Crude Oil
10 MPa	0.0292	0.0300
15 MPa	0.0231	0.0236
20 MPa	0.0219	0.0221
25 MPa	0.0216	0.0217
30 MPa	0.0204	0.0207
35 MPa	0.0205	0.0208

**Table 2:** Summary of initial miscible time (ns) in different systems.

	333 K	343 K	353 K	363 K	373 K
10 MPa	4.0	5.0	3.5	4.0	3.0
15 MPa	4.5	6.0	3.5	3.0	2.5
20 MPa	4.5	5.0	4.0	3.0	3.0
25 MPa	4.0	3.5	4.0	3.0	3.5
30 MPa	3.5	4.0	4.0	3.0	4.0
35 MPa	4.5	4.0	4.5	3.5	4.0

**Table 3:** Summary of MMP (MPa) obtained from CO_2_/crude oil in different systems.

	CO_2_	Crude Oil
333 K	20.31	20.21
343 K	21.08	20.89
353 K	22.12	22.36
363 K	24.43	23.84
373 K	26.25	24.52

**Table 4:** Summary of MMP (MPa) and relative error predicted by experimental and different empirical correlations.

Model	Number of Parameters	Predicted MMP (MPa)	Relative Error (%)
Yu et al. [51]	-	22.75	-
CO_2_ (this study)	1	19.63	13.71
Crude Oil (this study)	1	20.81	8.53
Lee [52]	1	20.84	8.32
Alston et al. [53]	4	19.72	13.22
Shokir [54]	8	20.03	11.89
Emera and Sarma [25]	2	30.11	32.44
Cronquist [55]	3	26.59	16.96
Glaso [56]	2	27.60	21.41
Yellig and Metcalfe [57]	1	16.55	27.18

## Data Availability

The data presented in this study is available upon reasonable request.

## Supplementary Materials

The following are available online. Figure S1: The number of crude oil atoms passing through the initial interface. Figure S2: Acquisition of MMP in different systems. Table S1: The number of CO_2_ molecules added in different systems. Table S2: Integrated values of CO_2_ and crude oil at different temperatures. Table S3: Summarization of some famous empirical correlations.

## Sample Availability

Samples of the compounds are not available from the authors.
