# Supplementary material for: Determination of Minimum Miscibility Pressure of CO2–Oil System: A Molecular Dynamics Study
Source: Molecules. 2021 Aug 17;26(16):4983. doi: 10.3390/molecules26164983 (PMC8401628; doi:10.3390/molecules26164983)

Table S1

The number of CO_2 molecules added in different systems.

	333 K	343 K	353 K	363 K	373 K
10 MPa	561	481	428	392	362
15 MPa	1166	980	830	720	640
20 MPa	1395	1274	1145	1032	925
25 MPa	1517	1421	1321	1229	1131
30 MPa	1597	1519	1436	1358	1275
35 MPa	1663	1594	1518	1448	1382

Table S2

Integrated values of CO_2 and crude oil at different temperatures.

(a) 333 K

Time (ns)	10 MPa		15 MPa		20 MPa		25 MPa		30 MPa		35 MPa	
	CO_2	Crude Oil	CO_2	Crude Oil	CO_2	Crude Oil	CO_2	Crude Oil	CO_2	Crude Oil	CO_2	Crude Oil
0.5	14.443	272.264	28.931	158.919	33.432	128.571	32.878	110.210	38.153	109.541	38.787	98.325
1.0	21.492	294.619	39.498	185.950	48.890	160.622	46.969	140.515	50.803	136.343	52.304	129.914
1.5	28.359	305.306	50.763	211.987	59.923	186.987	56.359	162.302	66.302	170.264	68.176	156.352
2.0	33.157	314.444	57.705	225.626	67.863	203.661	68.770	186.748	74.466	190.578	71.132	169.471
2.5	39.082	327.616	64.456	237.826	71.482	216.015	74.454	200.596	78.499	198.468	78.032	180.574
3.0	42.094	335.113	67.483	247.023	75.460	223.075	80.436	211.153	89.346	223.901	80.508	184.499
3.5	43.112	332.648	73.509	261.916	82.019	237.031	86.322	227.134	92.939	228.390	86.623	198.655
4.0	45.348	338.111	73.750	256.954	82.594	241.453	91.647	241.193	93.179	232.188	92.367	212.714
4.5	46.117	346.222	77.709	266.948	89.376	249.416	91.847	242.394	94.941	236.370	97.584	222.462
5.0	47.102	343.009	77.130	263.264	90.875	254.557	93.538	243.007	92.325	228.591	101.723	227.953
5.5	48.799	350.188	80.710	274.261	88.832	252.105	97.410	247.929	95.334	237.317	99.341	238.013
6.0	48.811	348.790	78.829	273.827	87.930	250.641	98.908	254.503	100.199	245.945	100.435	239.173
6.5	48.384	348.543	80.460	272.605	89.325	253.517	97.387	250.752	102.128	248.867	99.494	239.680
7.0	48.804	346.455	80.923	275.342	92.203	259.613	102.214	256.880	101.798	246.223	99.172	245.784
7.5	47.005	346.649	82.952	274.548	94.707	260.369	101.908	258.578	101.653	248.178	99.875	249.235
8.0	50.719	351.596	81.615	273.358	93.055	257.972	97.952	251.563	100.078	239.190	101.954	251.553
8.5	50.755	352.054	81.546	275.911	90.213	253.704	101.197	254.065	98.105	242.699	102.987	250.852
9.0	50.060	350.345	79.959	273.986	92.764	260.442	101.037	260.470	95.721	239.173	107.992	256.589
9.5	52.517	354.282	84.857	283.126	95.516	266.566	100.916	260.795	100.242	247.688	108.208	239.513
10.0	53.752	358.128	84.359	283.229	93.776	259.894	104.237	263.485	101.767	248.950	108.480	241.170

(b) 343 K

Time (ns)	10 MPa		15 MPa		20 MPa		25 MPa		30 MPa		35 MPa	
	CO_2	Crude Oil	CO_2	Crude Oil	CO_2	Crude Oil	CO_2	Crude Oil	CO_2	Crude Oil	CO_2	Crude Oil
0.5	9.074	290.130	21.401	176.381	31.383	136.704	34.279	122.637	32.218	106.767	33.117	99.583
1.0	15.130	308.378	35.204	210.260	47.194	180.808	46.635	156.025	47.745	139.635	46.682	132.063
1.5	21.399	319.247	44.938	234.297	55.826	199.892	59.359	186.618	61.465	176.167	56.508	152.474
2.0	28.407	332.406	53.483	252.039	62.943	217.888	71.253	210.674	72.511	196.256	70.641	182.538
2.5	31.078	341.763	56.822	255.173	71.291	233.104	79.114	226.230	76.715	206.282	78.696	200.705
3.0	33.829	347.076	61.942	269.311	76.320	243.602	81.335	228.749	81.058	218.887	85.258	211.027
3.5	35.681	347.157	66.782	277.060	76.289	249.509	88.238	244.197	87.964	227.477	90.967	224.343
4.0	39.567	357.790	65.641	279.345	80.411	256.177	90.381	246.877	90.888	239.222	95.851	234.164
4.5	44.117	365.759	70.431	284.334	84.516	263.872	91.820	248.197	91.730	235.055	93.432	231.601
5.0	46.099	372.195	69.170	281.458	80.411	253.330	92.813	257.732	92.564	236.602	96.466	239.237
5.5	45.131	370.122	71.125	290.068	82.275	254.428	96.027	260.440	95.699	246.298	97.390	243.812
6.0	44.894	368.475	74.681	292.178	83.478	259.084	95.221	263.963	95.120	245.172	99.185	244.041
6.5	43.398	361.123	73.986	296.979	84.784	262.730	99.575	267.199	95.932	242.796	102.766	250.360
7.0	43.104	364.076	74.499	292.854	88.540	273.075	98.076	264.375	97.992	250.438	105.167	253.317
7.5	43.955	367.485	73.092	296.819	86.457	268.137	95.204	260.682	100.329	255.534	103.508	252.944
8.0	44.503	368.082	76.151	302.365	89.237	268.049	95.424	262.818	101.959	260.702	103.434	251.217
8.5	46.483	373.051	74.050	293.946	86.282	261.652	90.880	253.776	99.743	254.441	103.469	251.351
9.0	43.127	362.749	74.894	297.197	90.116	275.434	90.776	254.005	100.968	254.264	105.072	253.969
9.5	44.195	375.224	77.167	301.548	91.938	278.280	97.964	262.618	101.384	257.275	104.251	252.405
10.0	43.669	369.369	76.905	296.149	95.245	282.528	94.998	256.929	101.487	257.473	102.721	246.992

(c) 353 K

Time (ns)	10 MPa		15 MPa		20 MPa		25 MPa		30 MPa		35 MPa	
	CO_2	Crude Oil	CO_2	Crude Oil	CO_2	Crude Oil	CO_2	Crude Oil	CO_2	Crude Oil	CO_2	Crude Oil
0.5	10.707	293.716	21.286	206.799	28.675	158.879	33.055	130.118	38.274	130.127	37.652	119.454
1.0	18.325	317.604	31.660	235.343	46.306	203.339	50.548	179.001	51.398	165.987	52.850	154.791
1.5	25.444	331.487	40.644	251.357	56.541	221.367	57.838	197.107	61.033	186.680	63.518	175.288
2.0	29.017	339.306	46.768	271.776	62.497	229.058	66.901	215.783	67.592	201.002	74.516	196.797
2.5	32.038	346.772	53.898	277.855	67.259	244.044	75.089	233.163	80.237	223.183	82.000	213.727
3.0	32.862	346.308	58.177	289.765	72.959	261.849	79.018	241.531	82.121	231.617	85.512	227.057
3.5	38.565	358.474	64.583	302.758	75.602	263.329	82.806	248.632	83.173	233.865	85.980	226.275
4.0	42.586	364.706	63.908	303.924	80.572	274.288	87.127	258.263	86.803	234.998	87.291	230.699
4.5	40.660	365.745	63.885	304.574	81.884	280.839	88.034	263.228	86.775	239.054	92.178	235.174
5.0	41.338	366.072	65.736	308.961	83.582	278.220	86.466	261.533	92.409	252.090	96.143	244.914
5.5	41.420	367.300	66.670	309.555	83.372	280.673	88.674	262.692	94.811	258.411	94.548	241.559
6.0	39.507	358.055	65.616	310.498	80.246	275.137	85.600	258.114	96.301	257.305	96.200	247.107
6.5	39.183	361.945	64.817	304.547	83.969	281.306	86.963	259.135	94.494	251.074	97.919	248.049
7.0	42.342	368.741	64.688	300.601	82.110	280.476	86.869	256.004	90.924	248.738	102.363	257.385
7.5	43.481	369.675	67.663	317.350	80.271	275.467	89.645	264.854	94.561	255.528	100.431	253.382
8.0	41.460	364.875	67.032	313.035	84.788	284.645	86.027	255.960	96.395	257.746	100.643	253.030
8.5	42.294	368.631	68.614	313.026	81.801	279.449	89.089	263.917	97.411	264.634	101.863	259.227
9.0	40.312	363.141	68.882	311.881	83.527	284.548	89.300	268.477	96.652	258.624	99.018	252.121
9.5	40.252	365.321	67.941	311.958	83.249	278.558	89.195	261.130	96.226	257.105	101.387	259.242
10.0	40.991	358.478	65.904	311.110	83.103	277.824	91.329	264.816	91.952	249.212	100.348	255.541

(d) 363 K

Time (ns)	10 MPa		15 MPa		20 MPa		25 MPa		30 MPa		35 MPa	
	CO_2	Crude Oil	CO_2	Crude Oil	CO_2	Crude Oil	CO_2	Crude Oil	CO_2	Crude Oil	CO_2	Crude Oil
0.5	11.637	306.066	21.052	226.975	28.877	174.392	32.535	149.440	35.853	137.635	34.505	119.206
1.0	17.321	322.371	33.705	269.815	39.458	205.106	45.337	178.542	49.228	172.678	51.225	164.458
1.5	25.057	339.370	38.737	279.308	53.867	238.995	55.879	205.739	60.141	193.698	61.031	186.845
2.0	27.992	343.298	45.167	290.492	59.509	250.595	66.168	228.339	69.018	214.662	74.393	208.000
2.5	28.979	349.787	53.527	301.764	66.037	267.331	70.978	236.343	78.557	234.855	79.481	222.452
3.0	29.543	347.322	57.494	313.025	74.967	279.431	75.274	245.778	80.365	236.896	84.040	232.187
3.5	31.366	348.783	56.601	306.512	73.542	278.255	72.717	242.584	83.752	242.318	89.485	246.299
4.0	35.198	357.739	56.862	317.525	77.539	287.607	74.898	247.232	80.884	240.200	89.922	245.807
4.5	36.076	361.367	55.867	308.317	75.829	287.470	73.302	245.976	85.407	244.215	95.033	251.845
5.0	35.866	360.127	59.467	318.396	74.428	283.139	77.786	246.161	81.179	241.140	94.775	256.753
5.5	36.936	361.012	60.566	315.441	75.186	285.148	79.735	252.419	84.150	250.052	91.823	246.592
6.0	36.489	364.989	56.934	314.819	75.742	284.551	81.443	260.699	83.086	242.518	98.775	262.599
6.5	36.001	358.845	58.483	314.019	76.893	282.973	82.483	263.999	85.668	249.433	96.332	259.756
7.0	35.443	354.730	58.110	316.137	76.251	283.185	81.136	260.149	88.121	252.279	98.054	258.506
7.5	35.263	356.386	59.241	318.735	76.617	287.044	84.219	269.756	86.788	251.058	97.496	258.338
8.0	35.729	360.932	61.098	323.295	75.075	285.757	85.907	267.132	90.641	256.911	97.239	258.147
8.5	34.531	358.104	57.369	320.012	78.705	290.147	85.204	272.535	87.145	250.177	98.803	262.596
9.0	35.408	363.076	57.441	316.237	76.358	290.237	82.726	266.286	88.404	253.861	95.452	254.173
9.5	35.934	364.823	55.219	309.036	75.638	288.617	86.991	273.507	91.566	256.558	95.913	256.339
10.0	38.791	362.688	56.595	317.682	77.549	287.370	82.476	261.229	89.637	258.466	97.197	259.106

(e) 373 K

Time (ns)	10 MPa		15 MPa		20 MPa		25 MPa		30 MPa		35 MPa	
	CO_2	Crude Oil	CO_2	Crude Oil	CO_2	Crude Oil	CO_2	Crude Oil	CO_2	Crude Oil	CO_2	Crude Oil
0.5	11.505	308.750	18.719	243.101	27.359	196.574	31.150	165.101	37.017	146.843	37.919	136.045
1.0	20.224	327.262	28.074	267.571	39.371	221.970	47.271	208.421	54.108	192.752	52.831	172.870
1.5	24.731	335.976	35.822	288.637	49.890	248.020	58.573	223.702	63.417	208.894	63.443	196.447
2.0	27.789	340.562	43.614	299.066	57.725	267.353	64.874	243.222	70.273	229.026	73.281	213.448
2.5	27.820	351.617	47.222	307.686	62.350	274.961	68.737	251.254	69.758	229.067	77.257	229.196
3.0	30.178	353.533	46.282	313.585	64.662	280.664	70.293	254.947	75.288	238.599	79.587	231.142
3.5	32.759	364.843	49.285	315.013	64.443	281.270	76.908	265.262	74.940	238.104	83.459	236.387
4.0	32.120	360.734	49.280	315.363	68.458	289.568	76.919	268.725	78.837	249.833	84.788	241.376
4.5	30.205	349.640	50.923	311.857	65.110	282.477	77.311	262.476	83.555	258.067	87.808	253.241
5.0	33.382	358.877	51.892	322.345	66.007	285.754	79.255	275.099	81.973	251.397	92.871	256.130
5.5	31.858	356.750	51.371	315.981	69.044	288.822	82.249	276.346	85.247	262.863	91.963	252.947
6.0	34.058	363.913	51.520	313.129	70.709	295.431	79.835	274.854	84.206	255.071	90.247	252.745
6.5	34.516	355.931	50.488	314.013	74.472	303.190	75.242	264.456	80.588	249.424	91.706	254.376
7.0	35.455	365.350	54.264	323.653	76.214	305.003	78.571	269.959	83.675	255.511	86.884	247.296
7.5	30.974	358.064	50.644	317.767	75.989	304.103	77.587	270.071	84.421	253.755	88.277	250.829
8.0	31.993	358.153	53.371	328.832	76.418	305.135	81.222	273.688	86.151	262.376	89.909	251.234
8.5	33.103	360.813	53.614	322.379	76.228	302.325	81.269	277.993	87.571	263.215	94.148	261.682
9.0	31.617	364.741	49.809	316.754	74.046	300.259	81.740	275.100	87.422	268.086	92.673	256.187
9.5	34.261	364.471	55.777	332.395	72.041	291.554	80.079	271.855	91.829	274.457	87.054	245.353
10.0	31.852	363.441	52.343	320.527	70.037	290.980	79.501	267.295	91.692	275.565	89.932	252.377

Table S3

Summarization of some famous empirical correlations.

Researcher	Empirical formula
Lee [52]	$MMP = 7.3942 \times 10^{b}; b = 2.772 - \frac{1519}{492 + 1.8T}$
Alston *et al.* [53]	$MMP = 6.056 \times 10^{-6} \times (1.8T + 32)^{1.06} \times {MW_{C5+}}^{1.78} \times (\frac{Vol}{Int})^{0.136}$
Shokir [54]	$MMP = -0.068616 \times z^{3} + 0.31733 \times z^{2} + 4.9804 \times z + 13.432; z = \sum_{n-1}^{8} z_n; z_n = A3_n x_n^3 + A2_n x_n^2 + A1_n x_n + A0_n$
Emera and Sarma [25]	$MMP = 0.474265308 - 0.0187974 \times MW_{C7+} + (278.6388 \times 10^{-11} \times {MW_{C7+}}^{3.023} \times e^{809.9 \times {MW_{C7+}}^{-1.189}}) \times (1.8T_R + 32)$
Cronquist [55]	$MMP = 0.11027 \times (1.8T + 32)^{Y}; Y = 0.744206 + (0.0011038 MW_{C5+}) + (0.0015279 Vol)$
Glaso [56]	$MMP = 810.0 - 3.404 M_{C7+} + (1.700 \times 10^{-9} {M_{C7+}}^{3.730} e^{786.8 {M_{C7+}}^{-1.058}}) T$
Yellig and Metcalfe [57]	$MMP = 12.6472 + 0.015531 \times (1.8T + 3.2) + 0.000124192 \times (1.8T + 32)^{2} - \frac{716.9427}{1.8T + 32}$

Figure S1

The number of crude oil atoms passing through the initial interface (a) and its first-order variance (b) with time evolution. (333 K, 10 MPa)

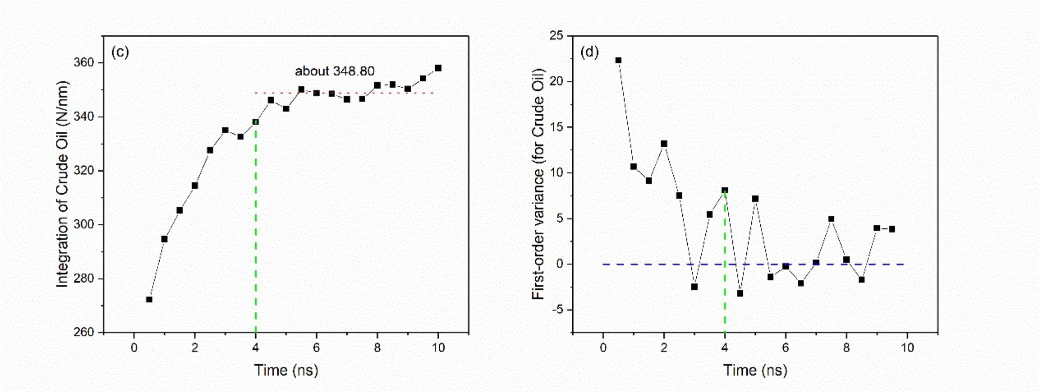

Figure S2

Acquisition of MMP in different systems.

343 K, for CO2 (c), for Crude oil (d).

353 K, for CO2 (e), for Crude oil (f).

363 K, for CO2 (g), for Crude oil (h).

373 K, for CO2 (f), for Crude oil (i).

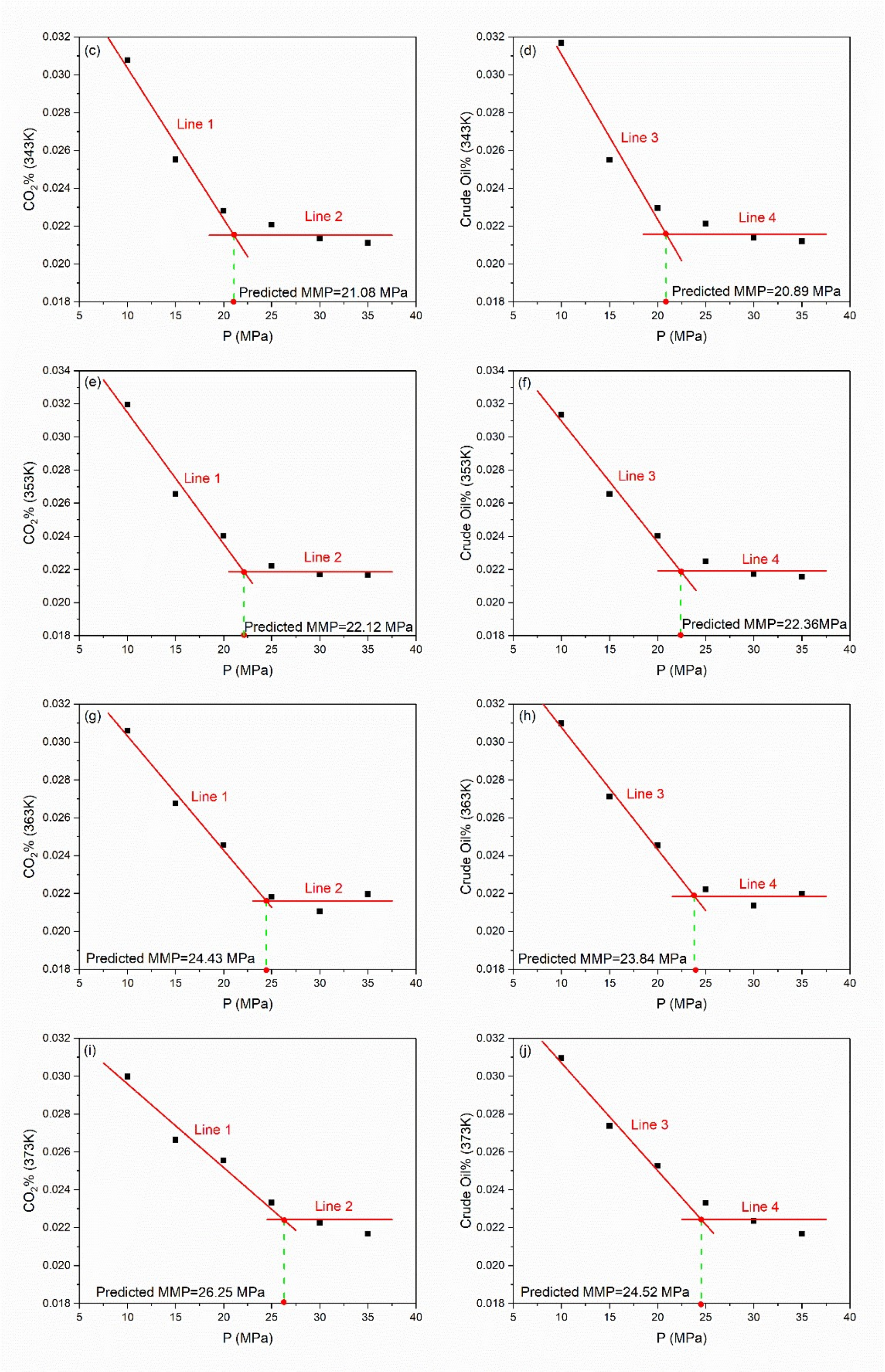

Supplement: Supplementary file 1 [file molecules-26-04983-s001.zip › molecules-1232886-supplementary.pdf]
